# Intermittend pneumatic venous thrombembolism (VTE) prophylaxis during neurosurgical procedures

**DOI:** 10.1007/s00701-024-06129-4

**Published:** 2024-06-14

**Authors:** Linda Oberle, Marcos Tatagiba, Georgios Naros, Kathrin Machetanz

**Affiliations:** Department of Neurosurgery and Neurotechnology, Eberhard Karls University, Hoppe-Seyler-Straße 3, 72076 Tuebingen, Germany

**Keywords:** Venous thromboembolism, Intermittend pneumatic compression, Pulmonary embolism, Neurosurgery, Deep venous thrombosis

## Abstract

**Background:**

The management of perioperative venous thrombembolism (VTE) prophylaxis is highly variable between neurosurgical departments and general guidelines are missing. The main issue in debate are the dose and initiation time of pharmacologic VTE prevention to balance the risk of VTE-based morbidity and potentially life-threatening bleeding. Mechanical VTE prophylaxis with intermittend pneumatic compression (IPC), however, is established in only a few neurosurgical hospitals, and its efficacy has not yet been demonstrated. The objective of the present study was to analyze the risk of VTE before and after the implementation of IPC devices during elective neurosurgical procedures.

**Methods:**

All elective surgeries performed at our neurosurgical department between 01/2018–08/2022 were investigated regarding the occurrence of VTE. The VTE risk and associated mortality were compared between groups: (1) only chemoprophylaxis (CHEMO; surgeries 01/2018–04/2020) and (2) IPC and chemoprophylaxis (IPC; surgeries 04/2020–08/2022). Furthermore, general patient and disease characteristics as well as duration of hospitalization were evaluated and compared to the VTE risk.

**Results:**

VTE occurred after 38 elective procedures among > 12.000 surgeries. The number of VTEs significantly differed between groups with an incidence of 31/6663 (0.47%) in the CHEMO group and 7/6688 (0.1%) events in the IPC group. In both groups, patients with malignant brain tumors represented the largest proportion of patients, while VTEs in benign tumors occurred only in the CHEMO group.

**Conclusion:**

The use of combined mechanical and pharmacologic VTE prophylaxis can significantly reduce the risk of postoperative thromboembolism after neurosurgical procedures and, therefore, reduce mortality and morbidity.

## Introduction

Surgical interventions increase the susceptibility to venous thromboembolism (VTE) due to surgical trauma, changes in blood flow dynamics, prolonged immobilization and activation of the coagulation system [4, 34]. During neurosurgical procedures, moreover, anticoagulants taken preoperatively are usually paused to minimize the risk of intraoperative or postoperative bleeding. This results in an additional risk of deep venous thrombosis (DVT), pulmonary embolism (PE) and subsequent complications in patients on long-term anticoagulation. Consequently, previous studies demonstrated a significantly increased incidence of both clinically symptomatic and non-symptomatic VTE in neurosurgical patients with a frequency of 9–16% asymptomatic DVT in doppler ultrasound screening [13, 15, 31].

General principles of perioperative prophylaxis of VTE contain a) pharmacological agents, (e. g., low-dose heparin or low molecular weight heparin (LMHW)), b) mechanical measures like compression stockings and intermittent pneumatic compression devices (IPC) or, c) multimodal prophylaxis with pharmacological and mechanical prophylaxis [5, 8]. Unfortunately, the management of peri-operative VTE prophylaxis is highly variable between neurosurgical departments and general guidelines are missing, especially for intracranial procedures [1, 9, 26, 32]. A representative survey of neurosurgeons in the United States found that most neurosurgeons use pharmacologic prophylaxis [1]. This is in line with study results from Germany, in which ~ 44% of neurosurgical departments started pharmacologic thromboprophylaxis not until the first postoperative day due to concerns about severe hemorrhagic complications [25]. In contrast, in orthopedics or general surgeries, it is recommended to start pharmacological prophylaxis with LMHW on the day of surgery [11, 22].

Only 27.2% of neurosurgeons use sequential compression devices [1]. However, IPC offers the opportunity to improve blood flow in the lower extremities by mimicking the natural muscle pump of the legs, thereby preventing blood stasis and clot formation without increasing the risk of intracranial hemorrhage [18, 19]. A few studies reported a reduction of postoperative VTE after neurosurgical interventions using IPC [24, 33]. Nevertheless, available data are scarce, general discussions of VTE prophylaxis focus on the dosage and timing of pharmacological prophylaxis, and there are no recommendations for the use of IPC or a combination of pharmacological prophylaxis and IPC.

In our neurosurgical department, combined mechanical and pharmacological thromboprophylaxis for well-defined treatment indications was introduced in April 2020 (Table 1). Previously, single-agent pharmacologic prophylaxis was administered beginning on the first postoperative day. The objective of the present study was to investigate the effect of combined IPC and pharmacological VTE prophylaxis compared to a single pharmacological therapy.

**Table 1:** Indications and contraindications for IPC

Intermittend pneumatic compression (IPC) in neurosurgery	
Indications	Contraindications
• Intracranial procedure > 2 h or spinal procedure > 2,5 h • Neoplasia (except pituitary adenoma) • Morbus Cushing’s disease • Paraparesis • Past history of VTE or hypercoagulation	• Current venous thrombosis, pulmonary artery embolism or thrombophlebitis • Decompensated heart insufficiency • Severe, uncontrolled hypertension • Acute soft tissue trauma of the extremities or compartment syndrome • Occlusion of the lymphatic vessels • Severe skin inflammation or disease in the treatment area (e.g. erysipelas) • Severe peripheral artery disease of the extremities

## Methods

### Patient characteristics

This retrospective, single-center study investigated all elective surgeries performed between January 2018 and August 2022 at the Department of Neurosurgery and Neurotechnology, Tuebingen, Germany, regarding the occurrence of VTE. The incidence of postoperative VTE before (CHEMO group; surgeries 01/2018–04/2020) and after (IPC; surgeries 04/2020–08/2022) implementation of mechanical thrombosis prophylaxis using IPC devices in April 2020 was compared. As approximately 3000 surgeries per year are performed in 4–5 operating rooms in Tuebingen, more than 12000 surgeries (CHEMO: *n* = 6663; IPC: *n* = 6688) were analyzed. Emergency procedures that result in a prolonged stay in the intensive care unit, such as aneurysm clipping due to subarachnoid hemorrhage, intracerebral hemorrhage, or decompression craniectomy for infarction were excluded. Furthermore, we excluded all patients with preoperative thrombosis. The study was approved by the local Hospital Ethics Committee and conducted in accordance with the declaration of Helsinki.

### Perioperative IPC management

**Fig. 1:**
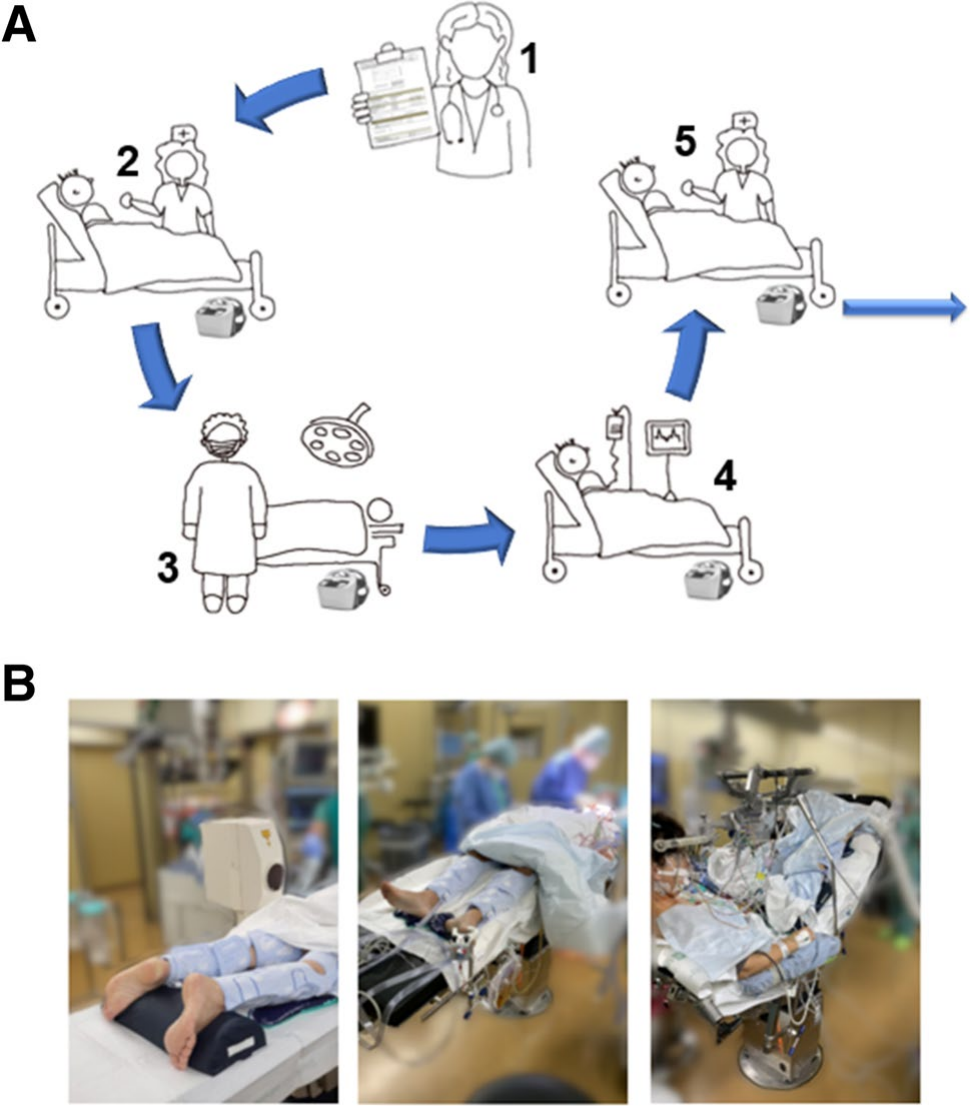
Illustration of IPC use in clinical routine: **A** Work-Flow of IPC Devices: physician indicates need for IPC, IPC stockings are applied by nursing stuff and remain on the patient during surgery and recovery room until the patient is transferred back to the neurosurgical ward; **B** Use of IPC devices in different positionings: prone, supine and semi sitting

Perioperative management and prophylaxis of venous thromboembolism was changed at the Department of Neurosurgery, Tuebingen, in April 2020. While previously a chemoprophylaxis with low-dose, low-molecular heparin (i.e., Clexane®, Sanofi-Avantis, Paris, France) was applied standardized for all patients subcutaneously from the first postoperative day until discharge (usually 20 mg/day after intracranial procedures and simple spine surgeries and 40 mg/day after complex spine procedures), an additional perioperative mechanical antithrombotic prophylaxis with IPC was now introduced for patients with increased risk profile for venous thrombosis. Therefore, the need for mechanical VTE prophylaxis was defined based on several criteria (Table 1). The indications and contraindications were defined as part of the development of a standard operating procedure (SOP) based on in-house experience and a literature review [7, 17, 27, 30]. If at least one criterion was fulfilled and there were no contraindications, IPC devices (Kendall SCD 700 Smart compression, Cardinal Health, Ohio, USA) were used peri-operatively until adequate mobilization of the patient was achieved, usually on the first post-operative day. The subsequent workflow was defined (Fig. 1A): After indication by the physician, the nurse applies the IPC devices at the neurosurgical ward on the morning of surgery. After transferring the patient to the operating room, compression of the lower extremities was started by the anesthesiologist preoperatively and continued throughout the surgery. The IPC devices were used in all surgical positions (e.g., concorde, semi-sitting, prone or supine positioning) (Fig. 1B). After surgery, the equipment and stockings were transported to the anesthesia recovery room/ Post Anaesthesia Care Unit (PACU) and finally to the neurosurgical ward while still attached to the patient. In case that none of the IPC criteria were fulfilled, only chemoprophylaxis was used for VTE prophylaxis. The product, timing or the amount of drug was not changed compared to the CHEMO group. Conventional antithrombotic compression stockings were not used in any of the groups.

### Financial impact of VTE

The German healthcare system uses Diagnosis Related Groups (DRG) according to the International Statistical Classification of Diseases and Related Health Problems classification (ICD-10) to calculate the costs of inpatient and day-care services. In this context, we analyzed the costs in cases with VTE in comparison to the mean costs of these ICD-10 codes annually published by our controlling (Tables 2 and 3). The cases were classified as malignant brain tumors, benign brain tumors, spinal surgery or trauma without prolonged ICU stay (e.g. chronic subdural hematoma). Group differences between IPC and CHEMO group were investigated.

**Table 2:** Characteristics of VTE patients. CHEMO: group with pharmacological thrombosis prophylaxis alone; DVT: deep venous thrombosis; IPC: group with mechanical and pharmacological thrombosis prophylaxis; PE: pulmonary embolism; VTE: venous thrombembolism

	Total	Chemo	IPC	
	*n* = 38/13351	*n* = 31/6663	*n* = 7/6688	X^2^ = 15.20 *p* < 0.001*
Age (years)	63.24 ± 13.15	64.2 ± 12.8	59.0 ± 14.94	t = 0.85 *p* = 0.209
Sex				
Male	24/38 (63.2%)	21/31 (67.7%)	3/7 (42.9%)	X^2^ = 1.520
Female	14/38 (36.8%)	10/31 (32.3%)	4/7 (57.1%)	*p* = 0.22
Type of thrombosis				
DVT	7/38 (18,4%)	4/31 (12.9%)	3/7 (42.9%)	X^2^ = 5.36
PE	29/38 (76.3%)	26/31 (83.9%)	3/7 (42.9%)	*p* = 0.068
PE + DVT	2/38 (5.3%)	1/31 (3.2%)	1/7 (14.3%)	
Time thrombosis after surgery (days)	5.53 ± 8.8	4.9 ± 8.7	8.4 ± 9.5	t = -0.911 *p* = 0.194
Death due to VTE				
No	34/38 (89.5%)	27/31 (87.1%)	7/7 (100%)	X^2^ = 1.009
Yes	4/38 (10.5%)	4/31 (12.9%)	0/7 (0.0%)	*p* = 0.315
Disease				
Malignant brain tumor	15/38 (39,47%)	12/31 (38.71%)	3/7 (42.9%)	X^2^ = 11.22
Benign brain tumor	11/38 (28,95%)	11/31 (35.48%)	0/7 (0.0%)	*p* = 0.011*
Spine surgery	8/38 (21.05%)	7/31 (22.58%)	1/7 (14,2%)	
Trauma	4/38 (10.53%)	1/31 (3.23%)	3/7 (42.9%)	
Preoperative paresis				
No	27/38 (71.1%)	22/31 (71.0%)	5/7 (71.4%)	X^2^ = 0.001
Yes	11/38 (28.9%)	9/31 (29%)	2/7 (28.6%)	*p* = 0.98
Duration inpatient stay (days)				
Overall	12.66 ± 9.42	14.03 ± 9.80	6.57 ± 3.64	*p* = 0.057
Malignant brain tumor	14.26 ± 9.55	16.25 ± 9.72	6.33 ± 0.58	*p* = 0.005*
Benign brain tumor	12.45 ± 12.59	12.45 ± 12.59	-	-
Spine surgery	11.88 ± 5.33	12.71 ± 5.15	6.00 ± 0.00	*p* = 0.269
Trauma	8.75 ± 6.18	14.00 ± 0.00	7.00 ± 6.25	*p* = 0.434
Total costs				
Overall	24,127.46 ± 20,568.94 €	24,205.47 ± 21,687.32 €	23,793.14 ± 16,279.34 €	*p* = 0.963
Malignant brain tumor	24,571.67 ± 13,435.53 €	25,456.57 ± 14,727.23 €	21,031.67 ± 6869.87 €	*p* = 0.628
Benign brain tumor	26,528.20 ± 30,050.96 €	26,528.20 ± 30,050.96 €	-	-
Spine surgery	19,156.13 ± 20,385.64 €	20,553.00 ± 21,601.51 €	9378.00 ± 0.00 €	*p* = 0.646

**Table 3:** Costs in VTE patients: The costs of patients with VTE (column 2) are shown in comparison to the average costs of all patients with the corresponding ICD-10 code (according to the DRG groups of the German health care system, column 3); ICD: International Statistical Classification of Diseases and Related Health Problems

	Costs of VTE patients (*n* = 38)	Mean costs of ICD-10 groups	Difference costs of VTE/mean costs ICD
ICD-10 groups			
Malignant brain tumor (C71)	24,571 €	16,342 €	8229 €
Benign brain tumor (D32 and D33)	26,528 €	12,730 €	13,798 €
Spine surgery (M48, M43.1 and M51)	19,156 €	8383 €	10,773 €

### Statistics and analyses

Evaluation and statistical analyses were performed using the SAP® software, SPSS (IBM SPSS Statistics for Windows, version 29.0; IBM Corp., Armonk/NY, USA) and MATLAB (MathWorks, Inc., Natick, MA, USA, R2022b). We analyzed the number of elective surgeries performed in the above-mentioned period. Subsequently an ICD-10 based automatic search was performed with the codes I26 for pulmonary embolism and I80 for venous thrombosis. In addition, cases were reviewed by two neurosurgeons. All cases with thrombosis or LAE based on emergency surgery with prolonged ICU stay were excluded from the analysis. The included cases were further used for statistical analysis in SPSS and classified into CHEMO and IPC groups. Group differences in distribution of clinical attributes such as sex, patients ‘ age, type of thrombosis, death due to VTE, duration between surgery and thrombosis, preoperative paresis, duration of inpatient stay or type of surgery (e.g., spine, brain tumor) were evaluated by Chi-squared or independent t-test. Statistical significance was considered at *p* < 0.05 for each statistical test.

## Results

### Patient characteristics

**Fig. 2:**
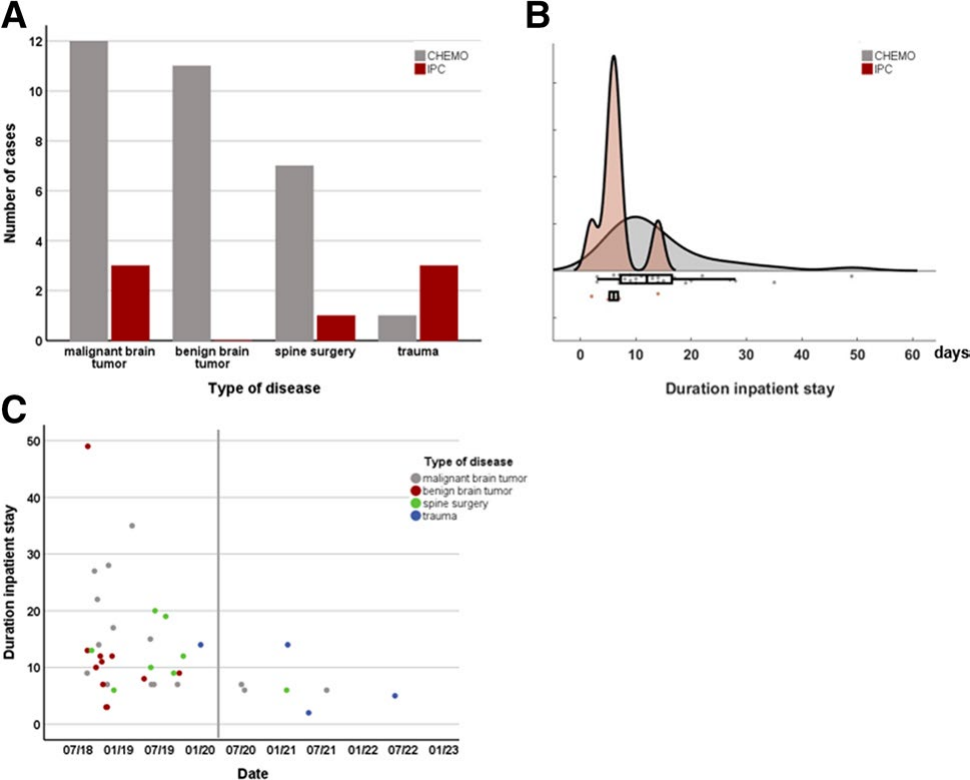
Illustration of VTE cases: **A** Number of cases in CHEMO and IPC for different disease groups (X2 = 11.22, *p* = 0.011*); **B** Raincloud plot of the inpatient stay in CHEMO and IPC in days; **C** Scatterplot of duration of inpatient stay for each individual case, red line indicates timepoint when IPC devices were established in the department

This retrospective study investigated 13351 neurosurgical procedures performed between January 2018 and August 2022. In total, 38 patients (0.28%, 24/38 male) developed postoperative VTE around 5.52 ± 8.8 days after surgery (Table 3). The most common underlying surgery prior to occurrence of VTE was for malignant brain tumors (15/38, 39.47%), followed by surgeries for benign brain tumors (11/38, 28.95%) (Fig. 2). While deep vein thrombosis of the legs could be detected in 7/38 (18.4%) and pulmonary artery embolism in 29/38 (76.3%), both deep vein thrombosis and pulmonary artery embolism could be diagnosed in 2/38 (5.3%) patients. Four of the patients died due to VTE.

### CHEMO vs. IPC

After introduction of IPC thromboembolism prophylaxis, significantly fewer of the surgically treated patients developed VTE (7/6688, 0.1%) than with CHEMO prophylaxis alone (31/6663, 0.47%) (X^2^ = 15.20, *p* < 0.001). There were no significant group differences (CHEMO vs. IPC) regarding age, sex, frequency of a preoperative paresis, and type or time of VTE appearance after surgery. Statistical analyses demonstrated a significant difference of the type of disease (X^2^ = 11.22, *p* = 0.011). In both, the CHEMO and IPC groups, patients with malignant brain tumors represented the largest proportion of patients with VTE (12/31 (38.71%) and 3/7 (42.9%)). However, while in the CHEMO group patients with benign tumor constituted 11/31 (35.48%) patients, the IPC group did not contain any patient with benign brain tumor. Although the difference in the frequency of death after VTE was not statistical significant between groups, it is nevertheless notable that none of the patients in the IPC group died, whereas 4/31 (12.9%) patients in the CHEMO group did. Similarly, the length of hospital stay of VTE patients in the IPC group was shorter than that in the CHEMO group, although not significantly.

### Financial impact of VTE

In the german health care system, DRG groups according to ICD-10 classification are used to calculate the costs and compensation for an inpatient stay. In this context, we could not demonstrate a significant difference in costs between IPC and CHEMO group. However, the mean costs of VTE patients were considerably higher than the mean costs of the same ICD-10 codes published by the controlling of our neurosurgical department (Table 3).

## Discussion

The management of postoperative VTE prophylaxis in neurosurgical departments still remains a highly diverse and controversial topic without general guidelines [6]. Different studies in the UK and the US have shown a clear consent for the need of post-operative VTE prophylaxis [1, 32]. However, opinions differ considerably regarding the correct method of prevention. In the current german guidelines on venous thromboembolism, IPC is recommended in addition to pharmacological prophylaxis or alone in case of contraindication for pharmacological methods in general surgery [11]. However, in neurosurgical settings mechanical VTE prophylaxis is yet not fully appreciated and seldom recommended as the evidence base for defining general prescriptions is scarce [2, 23, 35].

Pharmacological VTE prophylaxis is used effectively in many surgical disciplines 6–12 h after surgery [36]. However, it is associated with an increased risk of bleeding, which can be particularly life-threatening in cranial procedures and is therefore not generally used in neurosurgical patients before the first postoperative day. Adeeb et al. [1] reported that neurosurgeons with longer medical experience prefer mechanical compared to pharmacological VTE prophylaxis. This does not increase the risk of bleeding [14], but can be used already during the surgery. Since the peak of plasma concentration of the thrombin-antithrombin III complex occurs 3 h after the beginning of surgery, this may be a further advantage of IPC [12]. Previous studies have demonstrated the positive effect of IPC during neurosurgical surgeries [24, 33]. In line, our results demonstrated, that the introduction of combined mechanical (i.e., IPC) and pharmacological VTE prophylaxis leads to a significant reduction in the total number of postoperative VTEs in comparison to pharmacological prophylaxis alone. Furthermore, a reduction in the length of hospitalization – although not statistically significant in the overall cohort—may indicate a reduction in the severity of thromboses. This is particularly evident in patients with malignant brain tumors, who—consistent with previous studies indicating that the presence of malignant brain tumors increases the risk of VTE [10, 16, 20, 29]—had the predominant number of postoperative VTEs in both study groups. In literature, VTEs are described in 2–8% of cases after surgical resection of benign brain tumors (e.g., meningiomas) [21, 28]. Accordingly, in our study 11 patients of the CHEMO group with a benign tumor suffered from VTE. In contrast, no patient with a benign tumor developed VTE in the IPC group, while trauma patients accounted for 49.2% of VTE cases. These findings may indicate that combined pharmacological and mechanical prophylaxis with IPC can adequately prevent VTE in patients with benign intracranial tumors. However, no definitive statement can be made on the basis of the present results and further prospective and multivariate analyses are necessary to clarify these issues. In trauma patients (i.e., mostly chronic subdural hematoma), who according to the described indications and contraindications for IPC (Table 1) also in the IPC group did not received mechanical VTE prophylaxis, the indication for IPC should be reconsidered in the future. Although these are usually short surgeries (< 2 h), risk factors such as a prolonged postoperative immobilization (due to subdural drainage) or an increased age of these patients contribute to this. Further studies are needed in the future to better determine the indications for the use of IPC in neurosurgical patients.

Besides the medical benefit, the financial aspect must always be considered when introducing new technologies in clinical routine. The use of IPC involves the purchase of compression devices (reusable) and compression socks (disposable product), which initially increases the cost of combined IPC and pharmacological VTE prophylaxis compared to pharmacological prevention alone. However, since the costs of the hospital treatment are significantly increased by the occurrence of a VTE—as mentioned in our study—the reduction of VTEs can also lead to a reduction in costs. In order to determine the actual financial impact, future prospective studies should examine how many patients need to be treated (NNT) with combined prophylaxis to prevent thrombosis and how much money can be saved by this. Thus, the costs for IPC (per person) can be calculated in relation to the costs saved by preventing VTE.

### Limitations

Our study supports previous findings indicating a positive effect of IPC in neurosurgical patients [24, 33], but is limited by its retrospective nature and the lack of multivariate analysis of potential contributing factors and pre-existing risk factors for VTE. Therefore, we cannot exclude that factors such as e.g. sex, type of surgery, surgery time, mobilization postoperatively, diabetes mellitus or hypertension, have an impact in the altered rates of VTE in the comparison of both groups. This should be investigated in further prospective studies. Furthermore, the study did not investigate whether the combination of IPC with different chemoprophylaxis (e.g., LMWH or unfractionated heparin) leads to different outcomes, as indicated by an earlier review [3]. Finally, there was no systematic examination using doppler ultrasound, potentially missing patients with asymptomatic thrombosis.

## Conclusion

This study demonstrates that combined pharmacological and mechanical VTE prophylaxis is able to significantly reduce the occurrence of VTE in elective neurosurgical procedures compared to pharmacological prophylaxis alone. We believe that IPC is a safe and good tool for improving patient care during/after surgery. Besides the clinical benefit for the patient, IPC offers the opportunity to reduce costs by reducing VTEs. However, further studies and analyses must be conducted to evaluate the costs of purchasing and maintaining IPC devices compared to the financial gains from avoided VTEs.

## Data Availability

Data can be provided on reasonable request.

## Abbreviations

DRG: Diagnosis Related Groups
DVT: Deep venous thrombosis
ICD: International Statistical Classification of Diseases and Related Health Problems
IPC: Intermittend pneumatic compression
LMHW: Low molecular weight heparin
PACU: Post Anaesthesia Care Unit
PE: Pulmonary embolism
VTE: Venous thromboembolism
